# Protein-bound drugs are prone to sequestration in the extracorporeal membrane oxygenation circuit: results from an *ex vivo* study

**DOI:** 10.1186/s13054-015-0891-z

**Published:** 2015-04-14

**Authors:** Kiran Shekar, Jason A Roberts, Charles I Mcdonald, Sussan Ghassabian, Chris Anstey, Steven C Wallis, Daniel V Mullany, Yoke L Fung, John F Fraser

**Affiliations:** Critical Care Research Group, Adult Intensive Care Services, The Prince Charles Hospital and The University of Queensland, Rode Road, Chermside, 4032 Australia; Burns Trauma and Critical Care Research Centre, The University of Queensland, Herston, Queensland, Chermside, 4029 Australia; Centre for Integrated Preclinical Drug Development, The University of Queensland, Herston, Queensland 4029 Australia; Department of Critical Care Medicine, Nambour General Hospital, Nambour, 4560 Queensland Australia; Inflammation and Healing Research Cluster, School of Health and Sport Sciences, Faculty of Science, Health, Education and Engineering, University of the Sunshine Coast, Sippy Downs, 4556 Australia

## Abstract

**Introduction:**

Vital drugs may be degraded or sequestered in extracorporeal membrane oxygenation (ECMO) circuits, with lipophilic drugs considered to be particularly vulnerable. However, the circuit effects on protein-bound drugs have not been fully elucidated. The aim of this experimental study was to investigate the influence of plasma protein binding on drug disposition in *ex vivo* ECMO circuits.

**Methods:**

Four identical ECMO circuits comprising centrifugal pumps and polymethylpentene oxygenators and were used. The circuits were primed with crystalloid, albumin and fresh human whole blood and maintained at a physiological pH and temperature for 24 hours. After baseline sampling, known quantities of study drugs (ceftriaxone, ciprofloxacin, linezolid, fluconazole, caspofungin and thiopentone) were injected into the circuit to achieve therapeutic concentrations. Equivalent doses of these drugs were also injected into four polypropylene jars containing fresh human whole blood for drug stability testing. Serial blood samples were collected from the controls and the ECMO circuits over 24 hours, and the concentrations of the study drugs were quantified using validated chromatographic assays. A regression model was constructed to examine the relationship between circuit drug recovery as the dependent variable and protein binding and partition coefficient (a measure of lipophilicity) as explanatory variables.

**Results:**

Four hundred eighty samples were analysed. There was no significant loss of any study drugs in the controls over 24 hours. The average drug recoveries from the ECMO circuits at 24 hours were as follows: ciprofloxacin 96%, linezolid 91%, fluconazole 91%, ceftriaxone 80%, caspofungin 56% and thiopentone 12%. There was a significant reduction of ceftriaxone (*P* = 0.01), caspofungin (*P* = 0.01) and thiopentone (*P* = 0.008) concentrations in the ECMO circuit at 24 hours. Both protein binding and partition coefficient were highly significant, with the model possessing a high coefficient of determination (*R*^2^ = 0.88, *P* <0.001).

**Conclusions:**

Recovery of the highly protein-bound drugs ceftriaxone, caspofungin and thiopentone was significantly lower in the ECMO circuits at 24 hours. For drugs with similar lipophilicity, the extent of protein binding may determine circuit drug loss. Future clinical population pharmacokinetic studies should initially be focused on drugs with greater lipophilicity and protein binding, and therapeutic drug monitoring should be strongly considered with the use of such drugs.

## Introduction

Extracorporeal membrane oxygenation (ECMO) is establishing itself as a viable ultimate support therapy for patients with severe cardiorespiratory failure resulting from a variety of clinical conditions, and its scope continues to expand [1-3]. Patients on ECMO receive multiple drugs in an attempt to either reverse the underlying pathology or to minimise and/or treat complications. In venovenous ECMO, a high proportion of the native cardiac output is required to pass through the oxygenator to achieve adequate systemic oxygenation. In venoarterial ECMO, the ECMO circuit flows may exceed native cardiac output [4]. This transit of blood through the extracorporeal circuit may result in degradation and/or sequestration of circulating compounds, including administered drugs [5].

In addition, ECMO is associated with significant pharmacokinetic (PK) alterations [6], most important of which is an increased volume of distribution (V_D_) and decreased drug clearance (CL). From a PK point of view, the addition of an extracorporeal circuit that can sequester and/or degrade drugs during transit, as well as modulate their V_D_ and CL, which presents a significant challenge. The drug, device and disease factors affecting PK during ECMO are very difficult to characterise in a critically unwell patient, and, as such, laboratory-based research that mimics the clinical scenario [7] should be used to fully understand the complex mechanisms behind the PK alterations.

Drug factors such as protein binding and lipophilicity play a key role in their absorption, distribution, metabolism and excretion. Drugs are transported partly as unbound drug and partly reversibly bound to blood components such as plasma proteins and blood cells. The unbound drug then diffuses to extravascular or tissue sites, where the pharmacologic effects are observed. The dynamic relationship between unbound drug concentrations in the blood and tissue sites determines the overall efficacy of the drug. The distribution of the drug and its tissue penetration are determined mainly by the extent of protein binding, degree of ionization, and lipophilicity [8]. Lipophilicity is the main determinant of a drug’s permeability. It is traditionally assessed by measuring the drug distribution between immiscible phases of *n*-octanol and water. The ratio of a drug’s concentration in *n*-octanol and water is referred to as the *partition coefficient* (*P*), the logarithm of which (log *P*) is commonly used to describe the lipophilicity of therapeutic drugs [9].

ECMO circuits, by binding both circulating proteins and the drugs, can therefore significantly influence the PK of administered drugs in critically ill patients. However, most available data on disposition of drugs in ECMO circuits are derived from neonatal studies that have used older generation of ECMO circuits [6]. Data from these studies reveal significant sequestration of drugs in the ECMO circuit [6,10], with the extent of loss dependent upon their physicochemical properties, type and age of the circuit and the pumps used [11,12]. There are limited data from studies based on circuits used in adult patients. Even though lipophilicity of drugs is widely believed to be a major drug-related factor for circuit sequestration [5,11], the implication of protein binding on circuit disposition has not been fully elucidated.

To address this, we undertook drug disposition studies in contemporary ECMO circuitry in adult patients using an *ex vivo* model of ECMO. We hypothesised that lipophilic and protein-bound drugs are more prone to sequestration in ECMO circuits. We have previously reported results for five drugs (meropenem, vancomycin, fentanyl, midazolam and morphine [5]), which highlighted the role of drug stability and lipophilicity in determining circuit drug loss. In this article, we present results for another six study drugs (ceftriaxone, ciprofloxacin, linezolid, fluconazole, caspofungin and thiopentone) and describe the influence of protein binding on drug disposition in ECMO circuits.

## Materials and methods

Ethical approval was obtained from the Research, Ethics and Governance Unit, The Prince Charles Hospital, Metro North Hospital & Health Service, Brisbane, Australia (HREC/12/QPCH/90). Informed consent was not relevant, as no human subjects were enrolled in this study. The methods have been published previously [5,13], and therefore only a brief overview of the methods is presented here.

### Extracorporeal membrane oxygenation circuits

Four pulse life support (PLS) ECMO circuits were used. They consisted of Bioline tubing (Netafim, Fresno, CA, USA), a QUADROX D oxygenator and RotaFlow pump head (MAQUET Cardiopulmonary, Hirrlingen, Germany). A bladder reservoir (R-38; Medtronic, Minneapolis, MN, USA) was added to provide compliance to the circuit and allow multiple fluid sampling from the closed circuit. The circuits were primed with Plasma-Lyte P-148 (Baxter Healthcare, Toongabbie, Australia) and then exchanged with ALBUMEX 4 (human albumin 40 g/L; CSL, Parkville, Australia) and fresh human whole blood (less than 5 days old, mean volume 420 ± 52 ml, provided by the Australian Red Cross Blood Service). Porcine mucous heparin (Pfizer Australia, West Ryde, Australia) was added to the circuits (5,000 U).

The final volume of the pressurised circuit was 668 ± 1.7 ml. Activated clotting time was maintained between 220 and 250 seconds. The circuit flow rate, oxygen tension and temperature were kept between 4 and 5 L/min, between 150 and 200 mmHg and at 37°C, respectively, to maintain the pH of the circulating blood in the range of 7.20 to 7.55 by addition of carbon dioxide gas to the circuit or by modulating fresh gas flows. Fentanyl (20 μg), morphine (100 μg), midazolam (100 μg), meropenem (10 mg), vancomycin (40 mg), propofol (1 mg), dexmedetomidine (5 μg), thiopentone (20 mg), ceftriaxone (50 mg), linezolid (10 mg), ciprofloxacin (5 mg), fluconazole (10 mg) and caspofungin (5 mg) were injected postoxygenator as a single bolus. The drugs with known incompatibilities to study drugs (for example, gentamicin and ticarcillin/clavulanate) were excluded. These bolus doses were selected to produce concentrations similar to clinical concentrations. Larger doses were used for the drugs that exhibit high protein binding.

### Controls

Four polypropylene jars with tight caps were filled with 50 ml of fresh human whole blood, and 500 U of unfractionated heparin were added to the jars for anticoagulation. Fentanyl (1.5 μg), morphine (7.5 μg), midazolam (7.5 μg), meropenem (0.75 mg), vancomycin (3 mg), propofol (75 μg), dexmedetomidine (0.375 μg), thiopentone (1.5 mg), ceftriaxone (3.75 mg), linezolid (0.75 mg), ciprofloxacin (0.375 mg), fluconazole (0.75 mg) and caspofungin (0.375 mg) were added to the control jars after collection of baseline blood samples. These quantities were chosen to produce study drug concentrations similar to those achieved in the ECMO circuit. The jars were then placed in an incubator at 37°C and rocked continuously to ensure even distribution of the drugs.

### Blood sample collection

Postoxygenator blood was collected into lithium heparin tubes (5 ml) at baseline and at 2, 5, 15 and 30 minutes and 1, 2, 6, 12 and 24 hours after addition of the drugs to the circuit. Blood samples (5 ml) were also obtained from the control jars at time intervals identical to those of the circuit. All blood samples were stored on ice prior to centrifugation (10 minutes at 3,000 *g*), and the plasma was separated and stored in clean, prelabelled polypropylene cryogenic vials at −80°C until analysis.

### Measurement of drugs in plasma samples

A robotic online solid-phase extraction (SPE) Symbiosis Pharma system (Spark Holland, Emmen, The Netherlands) was used to extract the thiopental and thiopental-d5 (internal standard) from plasma samples. The SPE was conducted using a HySphere C18 cartridge (Spark Holland), and the analytes eluted from the cartridges were directly transferred to an XTerra MS C18 column (Waters, Milford, MA, USA). Mass spectrometry (MS) in electrospray ionization in negative mode (QTRAP 5500; AB SCIEX, Concord, ON, Canada) was used as the detector. The liquid chromatography and extraction methods used were created by Symbiosis Pro for Analyst (version 2.1.0.0; Spark Holland) and submitted to the MS controlling software (Analyst 1.6).

For antibiotic analysis, plasma sample aliquots (100 μl) were combined with an internal standard before protein precipitation by addition of trichloroacetic acid (ciprofloxacin) or acetonitrile (ceftriaxone, caspofungin, linezolid and fluconazole). Ceftriaxone supernatant was washed with dichloromethane prior to instrumental analysis. Ceftriaxone and ciprofloxacin were analysed on a Prominence high-performance liquid chromatography system (Shimadzu, Kyoto, Japan). Caspofungin, Linezolid and Fluconazole were analysed on a Nexera-8030+ ultra-high-performance liquid chromatography tandem MS (Shimadzu, Kyoto, Japan). All separations were performed by reverse-phase chromatography. Ciprofloxacin was detected by fluorescence, ceftriaxone by ultraviolet detection, and caspofungin, linezolid and fluconazole by triple-quadrupole MS. All assays were validated and conducted according to the US Food and Drug Administration guidance on bioanalysis [14].

### Statistical analysis

The data consisted of a longitudinal and correlated time series. For each drug in both the control and experimental (circuit) assays, the drug assayers were assumed to be independent of one another. For continuous data, normality was checked using a Shapiro-Wilk test. Non-normally distributed data were transformed. The data were analysed using a time series in a generalized linear model with a normal link function. The results are reported as the mean ± standard deviation (SD) for normally distributed data, median (interquartile range) for non-normal or categorical data and the proportion—either fractional or as a percentage—for binary data. For the purposes of analysis, all drug levels were referenced to the concentration of that drug at zero hours (baseline) and are reported as the percentage change from that baseline.

An ordinary (least-squares) regression model was constructed to examine the relationship between the two explanatory variables, protein-bound fraction (F_B_) and log partition coefficient (log *P*) and the outcome variable, fraction of drug remaining at 24 hours (F_C24_). Model coefficients and their 95% confidence intervals are reported. The final model was also tested for fit to the data (adjusted *R*^2^), and its residuals were examined for normality and homoscedasticity. Throughout, the level of significance was set at *P* <0.05. STATA™ software (version 12.0; StataCorp, College Station, TX, USA) was used for all analyses.

## Results

**Figure 1:**
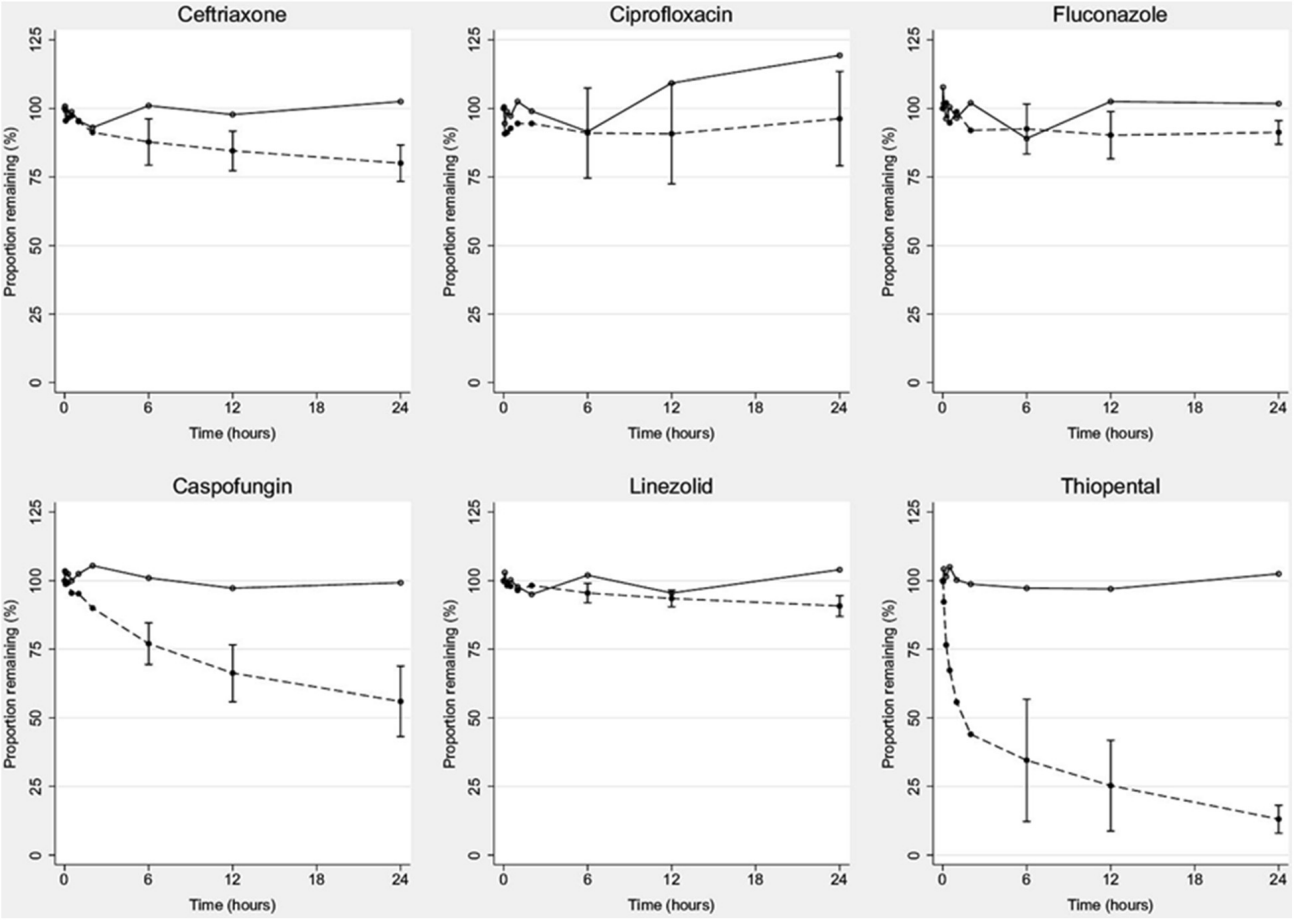
**Percentages of drug remaining in** ***ex vivo*** **extracorporeal membrane oxygenation circuits and the controls plotted against time.** For clarity, 95% confidence intervals are shown only for the experimental (circuit) group at times 6, 12 and 24 hours. The control group is identified by the continuous lines and open symbols. The circuit group is identified by the dashed lines and solid symbols.

The *ex vivo* circuits were maintained under physiological conditions for 24 hours without complications. The mean (SD) total protein and albumin concentrations in the circuits were 33 (2.5) g/L and 25 (0.9) g/L, respectively. The measured mean (SD) pH in the individual circuits over the 24-hour period were 7.20 (0.4), 7.33 (0.15), 7.39 (0.3) and 7.26 (0.14). A total of 480 samples (80 per drug) were analysed. The changes in drug concentrations relative to the baseline over time are summarised in Figure 1. Testing confirmed that all baseline plasma samples (prior to study drug injection into the circuit) were free of study drugs. There were no statistically significant differences in individual study drug recoveries between the four circuits or controls. The mean drug recoveries from the circuits and the control jars at 24 hours relative to baseline were, respectively, 80% and 102% for ceftriaxone, 96% and 119% for ciprofloxacin, 91% and 102% for linezolid, 91% and 102% for fluconazole, 56% and 99% for caspofungin, and 12% and 102% for thiopentone. The reduction in ceftriaxone (*P* = 0.01), caspofungin (*P* = 0.01) and thiopentone (*P* = 0.008) concentrations in the ECMO circuit at 24 hours were all significant. Although there was some variability in pH between circuits, there was no significant independent effect of pH on individual drug disposition in the circuits.

**Figure 2:**
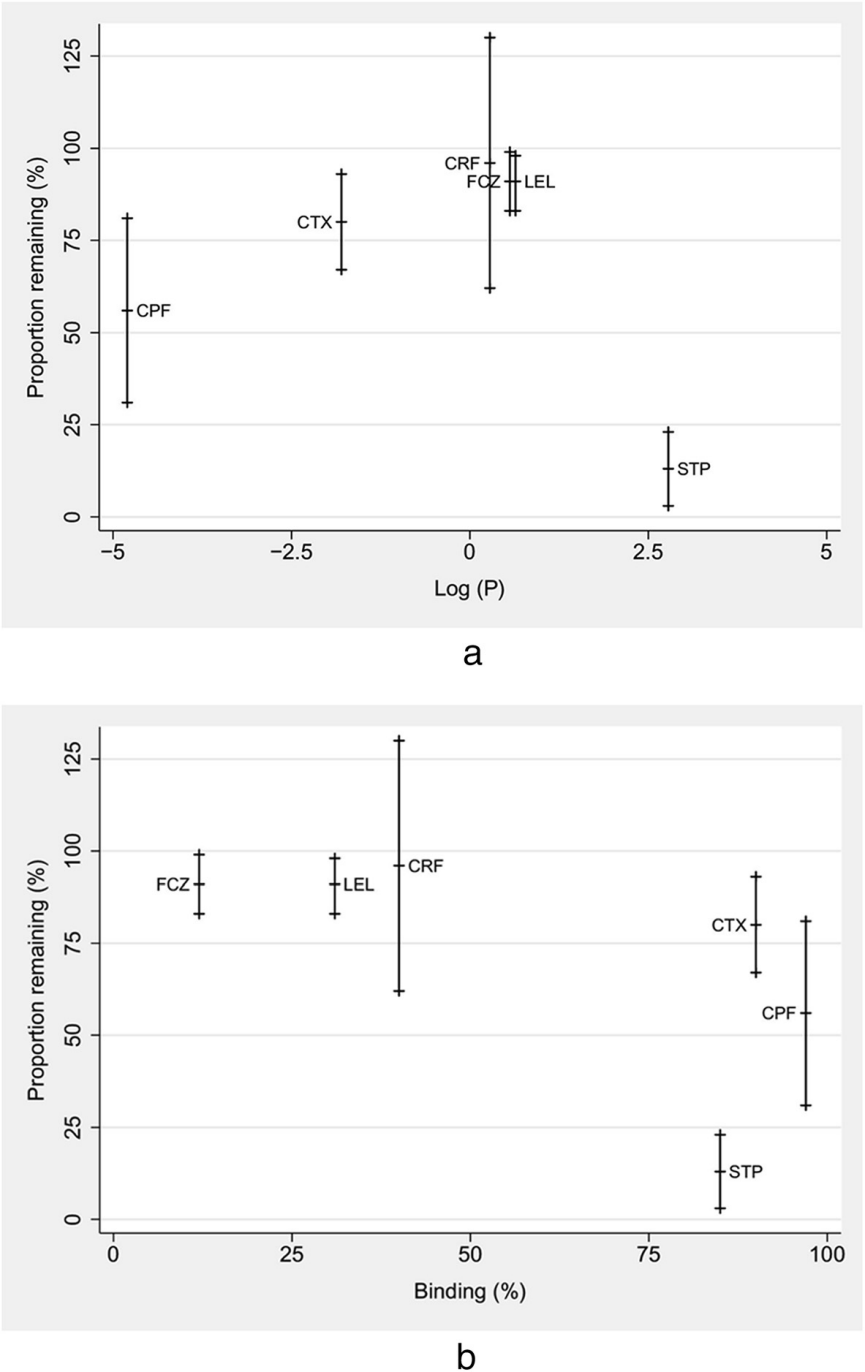
**Recovery of drugs in percent from extracorporeal membrane oxygenation circuit at 24 hours. (a)** Lipophilicity expressed as log partition coefficient (log *P*) values. **(b)** Protein binding expressed as percentage. For each drug, the mean concentration is indicated by a crossbar and the upper and lower 95% confidence intervals are indicated by crosses. FCZ, Fluconazole; LEL, Linezolid; CRF, Ciprofloxacin; STP, Sodium thiopentone; CTX, Ceftriaxone; CPF, Caspofungin.

Drug physicochemical data were obtained from the DrugBank online database [15] and then correlated with circuit drug behaviour. The relationships of study drug lipophilicity and protein binding with circuit drug concentrations are summarised in Table 1 and Figure 2. A linear regression model was constructed to examine the association between log *P* and the F_B_ in the prediction of F_C24_. In this model, both predictors were highly significant, with model diagnostics revealing normally distributed, homoscedastic residuals. The following model equation was used:

$$ {\mathrm{F}}_{\mathrm{C}24} = 1.21 - 0.17\  \log\ \mathrm{P} - 0.69\ {\mathrm{F}}_{\mathrm{B}}\left({R}_2 = 0.88\right). $$

The confidence intervals and associated *P*-values for the coefficients were as follows:

$$ \mathrm{Log}\ P\left[-0.17, - 0.13\right],P<0.001 $$

$$ {\mathrm{F}}_{\mathrm{B}}\left[-0.86, - 0.52\right],P<0.001 $$

**Table 1:** **Drug recoveries in** ***ex vivo*** **circuits and controls relative to baseline and their relationship to lipophilicity and protein-binding characteristics** ^**a**^

**Drug**	**Mean (SD) drug recovery (%) from controls at 24 hr**	**Mean (SD) drug recovery (%) from circuits at 24 hr**	**Lipophilicity (log** ***P*** **)**	**Protein binding (%)**
Ciprofloxacin	119 (4)	96 (17)	2.3	20 to 40
Fluconazole	102 (1)	91 (4)	0.4	12
Linezolid	102 (4)	91 (4)	0.9	31
Ceftriaxone	102 (1)	80 (6)	−1.7	95
Caspofungin	99 (8)	56 (13)	0.1	97
Thiopentone	102 (8)	12 (5)	2.3	80
Fentanyl*	82 (6.3)	3 (3.8)	3.9	85
Midazolam*	100 (3.6)	13 (2)	3.9	92
Meropenem*	42 (1.5)	20 (7)	−0.6	2
Vancomycin*	98 (9)	91 (11)	−3.1	55
Morphine*	103 (11)	97 (2.6)	0.8	30

^a^Log *P*, Log; SD, Standard deviation. Drugs that are significantly lost in the extracorporeal membrane oxygenation circuit are highlighted. Asterisks indicate previously published data from the same experiment [5].

## Discussion

This systematic investigation provides useful insights into the drug factors that influence drug disposition in circuitry currently used in adult patients undergoing ECMO. Based on a representative group of study drugs with diverse lipophilicity and protein-binding characteristics, this study demonstrates the importance of drug factors altering PK during ECMO. More important, the results indicate that for a given degree of lipophilicity, the extent of protein binding may determine circuit drug disposition. This is highly relevant, as the PK of highly protein-bound drugs are significantly affected during critical illness [16,17], and therapeutic drug monitoring (TDM) for many of these drugs is not routinely available at the current time.

The findings also highlight that there is considerable between-drug variability in the degree of drug sequestration. Drugs with significantly reduced concentrations at 24 hours were either highly protein-bound (>80%), highly lipophilic (log *P* >2.3) or both. As previously reported [5], meropenem (protein binding: 2%, log *P*: −0.6) was the only drug that did not conform to this trend, and its circuit loss can be attributed to its instability at physiological temperature [5,18]. Most other drugs that do not exhibit extremes of protein binding or lipophilicity remained relatively stable in the *ex vivo* ECMO circuit. Thus, drug stability at room temperature and at 37°C is also an important consideration for drugs prescribed during ECMO.

For a given solubility characteristic, the degree of protein binding appeared to be the main determinant of circuit drug concentration. For example, although ciprofloxacin and thiopentone have similar lipophilicity (log *P*: 2.3), greater reductions in 24-hour plasma concentrations were observed for thiopentone (88%), the more protein-bound drug as compared with ciprofloxacin (4%). For the hydrophilic drugs vancomycin and ceftriaxone (log *P*: −3.1 and −1.7, respectively), protein binding (55 and 83% to 95%, respectively) once again appeared to be the key determinant of circuit drug recovery (91% and 80%, respectively) at 24 hours.

The mean (±SD) total protein and albumin concentrations (33 ± 2.5 g/L and 25 ± 0.9 g/L, respectively) in the *ex vivo* circuit were quite similar to what is encountered in critically unwell patients [19]. As unbound study drug concentrations were not measured, it remains unclear whether protein-bound or -unbound fractions are more susceptible to circuit degradation and/or sequestration. In one study, there was a more significant loss of ampicillin (a relatively hydrophilic and less protein-bound drug) in neonatal *ex vivo* crystalloid-primed circuits [20] than in blood-primed circuits (72% vs. 15% lost at 24 hours). This indicates that the ECMO circuits can bind both proteins and drugs, and it is unclear if there is any competitive binding between them and, if so, whether such a phenomenon is concentration-dependent. Thus, the net circuit loss of a drug may represent a balance between binding to circuit components versus extent of protein binding. In addition, similar to their critically ill counterparts [16], patients receiving ECMO have physiological alterations that may influence protein binding, and a resulting increase in unbound drug fraction may enhance circuit losses [6]. This may, in part, explain the high V_D_ reported for drugs in patients receiving ECMO.

It is unclear if protein binding and lipophilicity have an additive effect on circuit drug sequestration, as some of the greatest decrements in circuit drug concentrations (>80%) reported at 24 hours [5] relate to drugs that have high degrees of both lipophilicity and protein binding (fentanyl, midazolam and thiopentone). This may be further substantiated by the fact that the less protein-bound drug ciprofloxacin (despite having a lipophilicity similar to that of thiopental) remained relatively stable in the circuit. The mechanisms that independently lead to circuit sequestration of a highly protein-bound drug are currently unclear. In a study using *ex vivo* neonatal circuits [21], up to 80% of the lipophilic and highly protein-bound drug fentanyl was lost in ECMO circuits without oxygenators at 6 hours, and addition of an oxygenator to the circuit only increased the losses by another 6%. It is possible that circuit sites that bind albumin and other circulating proteins upon priming or after passage of patients’ own blood may in turn bind to the administered drugs that exhibit high protein binding. Studies in which researchers have compared drug losses in clinically used versus new neonatal circuits have demonstrated significant variability in drug sequestration between the used and new circuits [11,12,22]. Consequently, it is still unclear if saturation of the drug-binding sites in the ECMO circuit over time occurs. Given that ECMO therapy may continue for many weeks, the time taken for saturation of both the protein- and drug-binding sites in the ECMO circuit also remains a subject for future studies. This could potentially be investigated with repeat dose experiments in a similar *ex vivo* model.

Studies in neonatal ECMO circuits have also demonstrated variable sequestration of drugs based on the different circuits, oxygenators and pumps used [11]. Even though these studies clearly identify lipophilicity as a factor for circuit drug sequestration, there are no published experiments that explore the impact of protein binding to the extent described in this study. Wildschut *et al*. [11] reported an 84% recovery for hydrophilic drug cefazolin (protein binding of 84%) at 3 hours in circuits with centrifugal pumps and polypropylene hollow fibre oxygenators. With silicone membrane oxygenators, the drug recoveries observed in blood-primed circuits by Mehta *et al*. for ampicillin, cefazolin and voriconazole were 85%, 79% and 29%, respectively. Although these three drugs exhibit contrasting degrees of lipophilicity (log *P*: −2, −1.5 and 1.0, respectively) and protein binding (25%, 84% and 58%, respectively), it should be noted that the least protein-bound and lipophilic drug of the three drugs—ampicillin—had the best recovery profile at 24 hours, despite its instability issues.

This *ex vivo* study has some limitations. The concurrent presence of several other physically compatible study drugs in the circuit and control jars mimicked the clinical scenario where patients receive these drugs concurrently, but it may have had an impact on competitive binding to blood proteins or the circuit components. Although there was some variability in pH between circuits, there was no significant independent effect of change in pH on individual drug disposition in the circuits, and similar drug loss trends were observed in all circuits. A reservoir bladder was necessary to allow removal of multiple blood samples from the otherwise non-compliant circuit, which may have influenced the circuit drug loss. Similarly, quantification of drug lost in control jars due to binding of drugs to the polypropylene container was not feasible, although this is suspected of being negligible because the surface area of the control experiment was significantly less.

The findings of this study may have significant implications for both the choice and the dosing of an individual drug prescribed during ECMO. Although any drug can be affected, these findings will inform the design of future clinical PK studies [23] that are the next logical step in the evaluation of the impact of the circuit and drug factors on PK in critically unwell patients receiving ECMO and in the development of robust dosing guidelines. Given that most of these drugs are highly relevant for this patient population, TDM, where available, is also strongly recommended, pending clinical PK data.

## Conclusions

This *ex vivo* study highlights the role of the ECMO circuit and drug factors in altering PK during ECMO. In addition to previously identified drug factors such as instability and lipophilicity, this study highlights the influence of protein biding on drug disposition in ECMO circuits. The drugs that are most significantly affected need expedited evaluation in clinical population PK studies and in further mechanistic studies in animal models so that the *in vivo* impact of such circuit drug losses are fully elucidated. Such mechanistic and clinical PK data can then assist the development of meaningful dosing simulations and robust dosing guidelines for the prescription of antibiotic and sedative drugs given during ECMO.

## Key messages

- Drug stability, lipophilicity and protein binding are the three key drug factors that influence drug disposition in ECMO circuits.
- Protein-bound drugs appear to be more significantly sequestered in *ex vivo* ECMO circuits.
- When multiple drugs with similar degrees of protein binding are administered, circuit drug loss is determined by degree of lipophilicity and vice versa.
- Sequestration of drugs in the circuit may have implications on both the choice and dosing of a particular drug prescribed during ECMO.

## Abbreviations

CL: Clearance
ECMO: Extracorporeal membrane oxygenation
F_B_: Protein-bound fraction
F_C24_: Fraction of the drug remaining in the circuit at 24 hours
Log *P*: Log partition coefficient
PK: Pharmacokinetics
PLS: Pulse life support
SD: Standard deviation
SPE: Solid-phase extension
TDM: Therapeutic drug monitoring
V_D_: Volume of distribution
